# Stabilized High Clay Content Lateritic Soil Using Cement-FGD Gypsum Mixtures for Road Subbase Applications

**DOI:** 10.3390/ma14081858

**Published:** 2021-04-08

**Authors:** Phattharachai Maichin, Peerapong Jitsangiam, Toon Nongnuang, Kornkanok Boonserm, Korakod Nusit, Suriyavut Pra-ai, Theechalit Binaree, Chuchoke Aryupong

**Affiliations:** 1 Department of Civil Engineering, Faculty of Engineering, Chiang Mai University, Huai Kaew Road, Mueang, Chiang Mai 50200, Thailand; phattharachai_mai@cmu.ac.th (P.M.); toon_n@cmu.ac.th (T.N.); 2 Center of Excellence in Natural Disaster Management, Department of Civil Engineering, Faculty of Engineering, Chiang Mai University, Huai Kaew Road, Mueang, Chiang Mai 50200, Thailand; cendru1@gmail.com; 3 Department of Applied Chemistry, Faculty of Sciences and Liberal Arts, Rajamangala University of Technology Isan, Nakhon Ratchasima 30000, Thailand; Kornkanok.bo@rmuti.ac.th; 4 Centre of Excellence on Energy Technology and Environment, Department of Civil Engineering, Faculty of Engineering, Naresuan University, Tha-Po, Mueang, Phitsanulok 65000, Thailand; korakodn@nu.ac.th; 5 GWR Research Unit, School of Engineering, University of Phayao, Phayao 56000, Thailand; suriyavut.pr@up.ac.th; 6 Department of Mechanical Engineering, Faculty of Engineering, Chiang Mai University, Huai Kaew Road, Mueang, Chiang Mai 50200, Thailand; theechalit_b@cmu.ac.th

**Keywords:** clay–cement stabilization, lateritic soil, FGD gypsum, unconfined compressive strength, SEM

## Abstract

With a lack of standard lateritic soil for use in road construction, suitable economical and sustainable soil-stabilization techniques are in demand. This study aimed to examine flue gas desulfurization (FGD) gypsum, a by-product of coal power plants, for use in soil–cement stabilization, specifically for ability to strengthen poor high-clay, lateritic soil but with a lower cement content. A series of compaction tests and unconfined compressive strength (UCS) tests were performed in conjunction with scanning electron microscope (SEM) analyses. Therefore, the strength development and the role of FGD gypsum in the soil–cement–FGD gypsum mixtures with varying cement and FGD gypsum contents were characterized in this study. The study results showed that adding FGD gypsum can enhance the strength of the stabilized substandard lateritic soil. Extra FGD gypsum added to the cement hydration system provided more sulfate ions, leading to the formation of ettringite and monosulfate, which are the hardening cementitious products from the cement hydration reaction. Both products contributed to the strength gain of the soil–cement–FGD gypsum material. However, the strength can be reduced when too much FGD gypsum is added because the undissolved gypsum has a weak structure. Examinations of FGD gypsum in the soil–cement–FGD gypsum mixtures by SEM confirmed that adding FGD gypsum can reduce the cement content in a soil–cement mix to achieve a given UCS value.

## 1. Introduction

**Figure 1:**
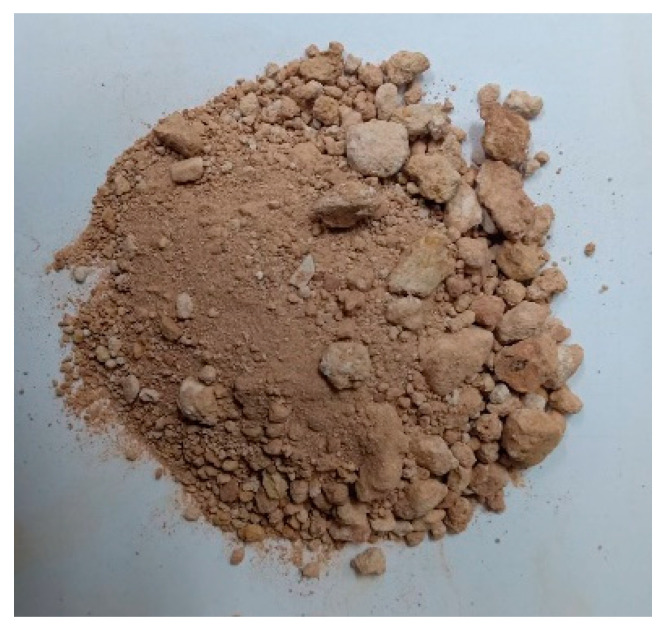
Lateritic soils.

Lateritic soil is iron- and aluminum-rich and formed in a special environment of relatively high temperature and humidity in a tropical area (Figure 1). It is formed from the weathering process of the parent rock, the laterization process, which begins with the removal of silica (SiO_2_) and the accumulation of some oxides from the parent rock, such as iron oxide (Fe_2_O_3_) and aluminum oxide (Al_2_O_3_) [1,2]. Lateritic soil is defined as having a ratio of SiO_2_/Al_2_O_3_ between 1.33 and 2.00 [3] and is noticeably reddish or brownish-red with a high clay content [4]. Such soil is also generally used in most tropical countries as a road construction material [5] because of its ready availability and relatively low cost. Furthermore, good lateritic soil has some satisfying engineering properties, and material specifications for the quality control of lateritic soil for road construction have been established (Table 1) [6,7,8,9,10,11]. However, over the past decade a shortage of standard natural road materials, including standard lateritic soil, has become a significant problem for the road construction industry. High-quality materials complying with the specifications established by road authorities are in short supply. Most of the available natural materials do not meet the standards for road construction because they have undesirable characteristics of high plasticity and substandard grading [12,13,14,15]. Consequently, soil improvement techniques, especially soil–cement stabilization, have become the new method for overcoming the scarcity of standard materials by improving the essential properties of poor soil to the level needed for practical use in road construction.

Soil improvement for road construction can be achieved by using techniques such as preloading, soil replacement, using recycled concrete aggregates, and using soil stabilizing agents [16,17,18,19,20]. The most popular technique is the use of ordinary Portland cement (OPC) as a stabilizing agent, leading to the technique of “soil–cement stabilization” [21], the goal of which is to increase soil strength. Some previous research [22,23,24,25,26,27,28] confirmed that using OPC to stabilize different soil types, including soft clay and lateritic soil, can improve their unconfined compressive strength (UCS). Furthermore, those UCS values increase as cement content and curing time increase. When this OPC clinker (Table 2) chemically reacts with water, the cementitious compounds of C–S–H (calcium silicate hydrate) and others are produced, resulting in a strength gain characteristic of stabilized soil. In addition, in Table 2, the presence of calcium sulfate dihydrate (gypsum) of approximately 5% by weight, can play a role in delaying the hydration reaction of calcium aluminates (C_3_A) in the OPC clinker [29,30,31] when reacting with water. This retarding effect caused by the gypsum in OPC could enhance the strength and durability of the cementitious products. However, even though OPC is one of the most popular construction materials at present, its production process and application generate carbon dioxide (CO_2_). The high CO_2_ footprint of OPC and OPC-oriented material production could profoundly affect global warming [32,33,34]. From a sustainability point of view, OPC would not be an environmentally friendly construction material. Moreover, for soil stabilization, OPC is relatively costly compared to the cost of the soil. Some recent research used only waste materials without OPC, such as rice husk ash [35], palm oil fuel ash [36], calcium carbide residue [37], or even waste beverage cans [38] that can be applied to improve soil properties. These waste materials have played a gradually more prominent role by avoiding cement consumption, which is the main problem with CO_2_ emissions. However, these materials will not be widely acceptable without OPC. For reasons of sustainability and cost, the development of cement substitutions for various construction applications is an active research field. There is potential to use industrial by-products, such as coal ash and furnace slag, or waste materials instead of OPC substitution. These materials maintain or increase the soil’s material strength while reducing the amount of OPC and the CO_2_ it generates [39,40,41,42].

**Figure 2:**
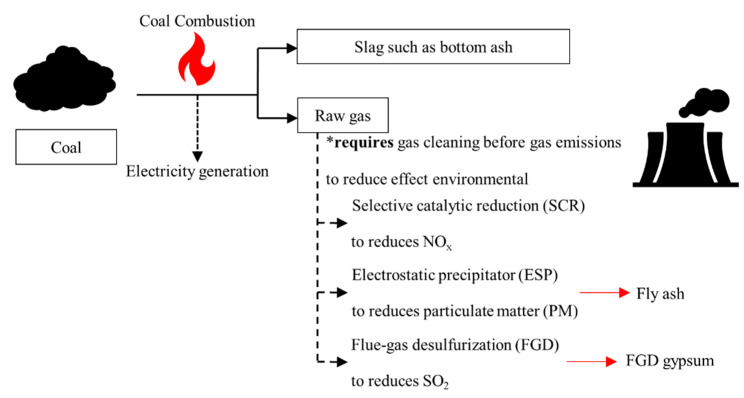
Pollution-control systems used to clean emissions from a coal-fired power plant.

**Figure 3:**
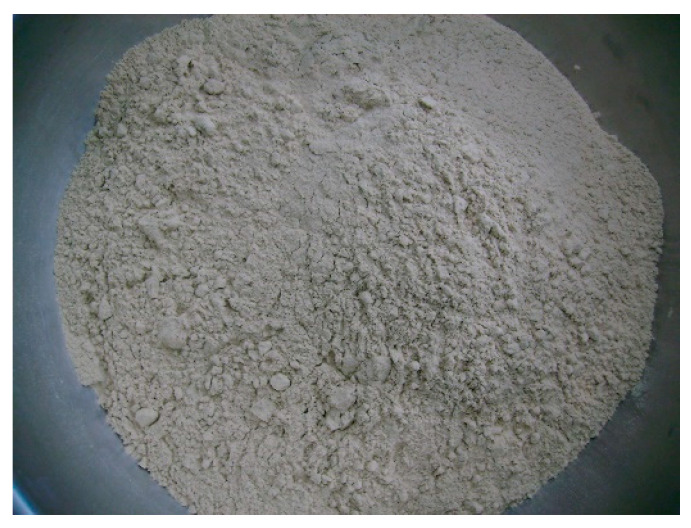
Flue gas desulfurization gypsum (FGD gypsum).

Coal-fired power plants generate electricity in many countries around the world. Based on their electricity generation (Figure 2), the flue gas desulfurization (FGD) plant plays a vital role in reducing sulfur dioxide (SO_2_) emissions [43,44]. This minimizes adverse environmental effects. Consequently, a by-product called FGD gypsum (CaSO_4_·2H_2_O) is generated in abundance (Figure 3). Its properties, including physical and chemical properties and mineral composition, are nearly identical to those of natural gypsum (Table 3) [43,45,46,47]. FGD gypsum has been used for specific applications such as landfills and agriculture [48,49]. The American Coal Ash Association (ACAA) reported that the U.S. produced around 23 million tons of FGD gypsum in 2019, with a 50% rate of reuse [50]. In Europe, the European Coal Combustion Products Association reported FGD gypsum production of approximately 10 million tons in 2016 [51]. Therefore, FGD gypsum is an industrial by-product that could be used more effectively. Using it as fill or road construction material would be a high-volume usage.

**Figure 4:**
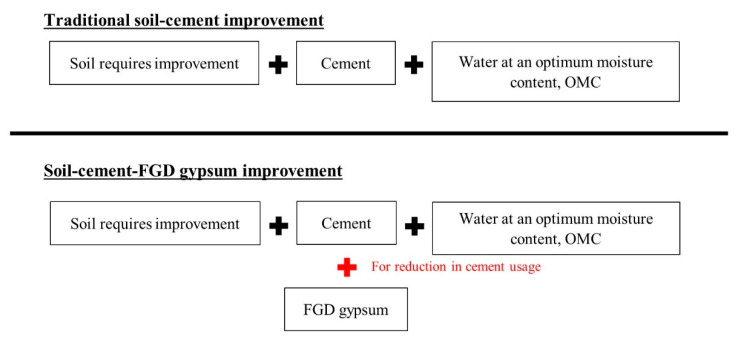
Traditional soil–cement process and the soil–cement–FGD gypsum process.

For soil–cement stabilization, FGD gypsum can be used as a cement substitute based on the additional gypsum concept. It can decrease the cement content and increase the compressive strength of the soil–cement material [52,53,54,55]. An optimum amount of gypsum in the soil–cement system is crucial because too much additional gypsum can cause left-over gypsum, which then becomes the weak element in the soil–cement material, referred to as “supersaturated gypsum”. FGD gypsum would be a viable cement substitute for soil–cement stabilization. Moreover, water, OPC, and FGD gypsum are essential parameters for the design mixture. Still, there are no clear guidelines on designing mixtures for soil stabilization when using stabilizing agents of more than one type. Therefore, in this study the relationship of those parameters has been established for soil–cement–FGD gypsum application. A soil–cement–FGD gypsum mixture is proposed in this study and is compared to the traditional soil–cement process (Figure 4).

This study aimed to examine the use of a soil–cement–FGD gypsum mixture for improving substandard lateritic soil with a relatively high clay content for the purpose of road construction. This research could be a guideline for using FGD gypsum in other soil–cement applications. In this study, a series of modified compaction tests [56] and UCS tests [57] were performed in the laboratory on the soil–cement–FGD gypsum mixtures with varying cement content (1–3% by dry weight), varied FGD gypsum content (1, 3, and 5% by dry weight), and curing periods (3, 7, and 28 days). In addition, California Bearing Ratio (CBR) tests [58] were carried out on soaked and unsoaked forms of selected mixes to establish the CBR index (%), the percentage between the CBR values of the soaked sample and the unsoaked sample. This CBR index was used to evaluate the moisture sensitivity of mixtures and soil. Microstructure observations through the scanning electron microscope (SEM) analyses were also performed to examine chemical interactions in a soil–cement–FGD gypsum matrix. Finally, a process to determine an optimal cement–FGD gypsum content corresponding to a target compressive strength was proposed.

## 2. Materials and Methodology

### 2.1. Materials

#### 2.1.1. Lateritic Soil

**Figure 5:**
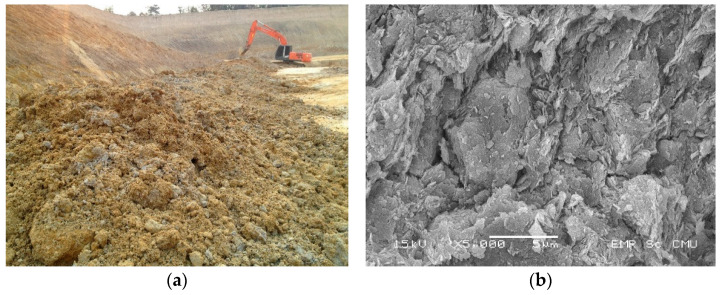
Lateritic soil (**a**) in overview; (**b**) microstructure.

**Figure 6:**
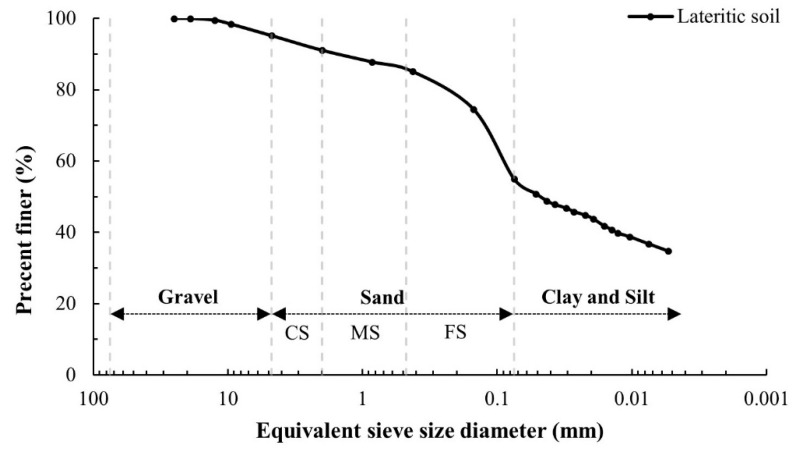
The grain size distribution of lateritic soil C.

Lateritic soils A, B, and C were sourced from three local areas in Chiang Mai, Thailand. The physical and engineering properties of those soils were measured in accordance with the standards set by the Department of Highway (DOH), Thailand, for use as a subbase material for the DOH [6]. All the lateritic soil samples had relatively high clay content with plastic index (PI) values higher than 11, the specified standard value for road construction (Table 4). These elevated PI values confirmed that the majority of the lateritic soils were substandard. As a result, those soils had to be modified and improved before they could be used as road construction material. In this research, substandard lateritic soil from location C (Figure 5a) was selected for use in laboratory tests because it had the highest PI value, which indicated the most substandard condition compared to the other two sources in this study. Hexagonal plates of clayey soil particles stacked into some spaces between the soil particles were observed in the SEM image of the lateritic soil from C (Figure 5b). This lateritic soil has a grain-sized distribution (Figure 6) and can be classified as low plasticity clay (CL) according to the Unified Soil Classification System (USCS) [59].

#### 2.1.2. Stabilizing Agents

A commercial type of Portland Cement (Type 1) complying with ASTM C150 [60] was used.

**Figure 7:**
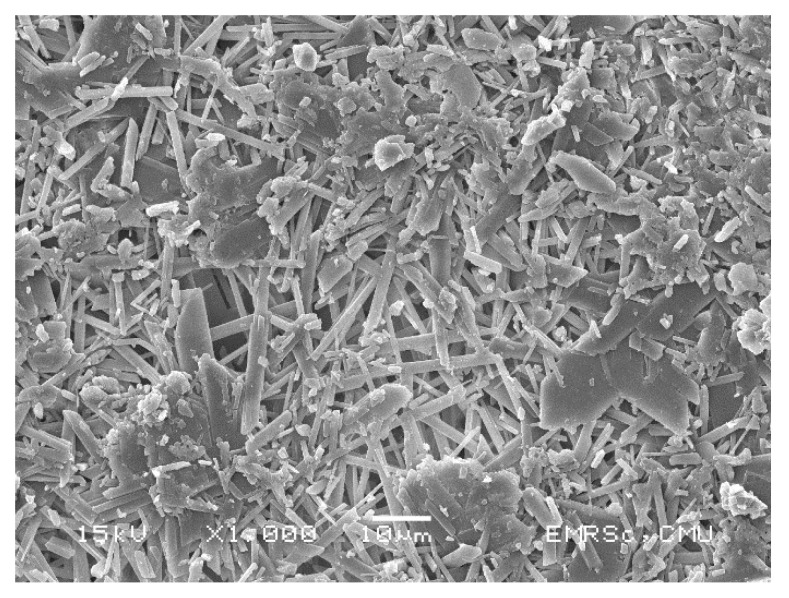
The microstructure of FGD gypsum.

FGD gypsum was collected from the Mae Moh coal power plant, Lampang, Thailand, where around 2.25 million tons of FGD gypsum is produced annually [61]. Figure SEM analysis of the FGD gypsum used in this study shows rod-shaped particles with sizes between approximately 5 and 20 µm (Figure 7).

### 2.2. Methodology and Experimental Works

#### 2.2.1. Test Standard

The experiments to determine the physical properties of lateritic soil and the strength development of soil–cement–FGD gypsum mixtures are detailed as follows:

*Physical properties*
  - Specific gravity tests in accordance with ASTM D854 [62];
  - Soil particle size distribution by wet sieving tests in accordance with ASTM D422-63e2 [63];
  - Soil particle size distribution by hydrometer tests in accordance with ASTM D422-63e2 [63];
  - Liquid limit tests and plastic limit tests in accordance with ASTM D4318 [64].
*Strength development of soil–cement–FGD gypsum mixtures*
  - Modified Proctor compaction tests in accordance with ASTM D1557 [56];
  - Unconfined compressive strength (UCS) tests in accordance with ASTM D1633 [57];
  - California Bearing Ratio (CBR) tests in accordance with ASTM D1883 [58];
  - Microstructure analyses by a scanning electron microscope (SEM), JEOL JSM-5910LV (JEOL Ltd., Tokyo, Japan).

#### 2.2.2. Methodology

**Figure 8:**
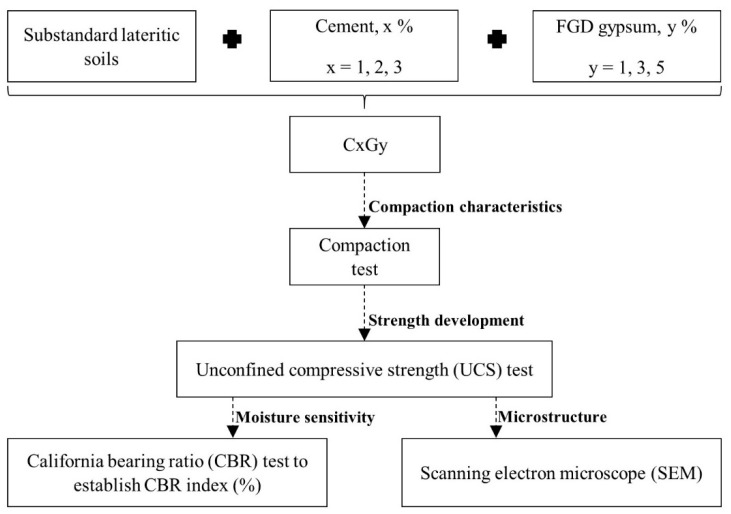
Methodology process of this research.

The study process started with the determination of the optimum moisture content (OMC) and the maximum dry density (MDD) values of all mixtures in this study using the modified Proctor compaction tests [56]. For each mix, the densification at the OMC and MDD was used to prepare test samples for UCS tests [57]. Next, UCS tests were performed to assess the strength development of the soil–cement–FGD gypsum mixtures. CBR tests [58] were also carried out on selected mixes for both soaked and unsoaked conditions, and then the CBR index (%) was established (see Equation (1)) to measure the moisture sensitivity of selected mixtures and soil indirectly. Lastly, the test samples of the highest compressive strength in each cement group were subjected to the microstructure observation through the SEM analyses (Figure 8). Based on a series of test samples in this study, the sample symbols were specified as CxGy, where *x* is the percentage amount of cement by dry weight soil, and y is the percentage amount of FGD-gypsum by dry weight soil.

CBR index (%) = (Soaked CBR Value/Unsoaked CBR value) × 100(1)

#### 2.2.3. Sample Preparation and Testing

The UCS tests by ASTM D1633 (Method A) [57] were followed based on cylindrical samples of size 101.6 mm in diameter and 116.4 mm in height. UCS test samples were compacted in five layers for 25 blows per layer to achieve their MDD values as target densities. After that, each sample was removed from the Proctor Mold and wrapped in a plastic bag to prevent moisture loss during the curing period and kept in at ambient temperature until the target curing periods of 3, 7, and 28 days were reached. Then, the UCS samples which had the highest UCS values were selected to observe the micro-interactions of the mixture matrix using the SEM analyses. The CBR tests by ASTM D1883 [58] were conducted on samples having the same mixture condition as those with the highest UCS values at a 28-days curing time. The substandard lateritic soils were stabilized by mixing OPC (C) in the proportions of 0, 1, 2, and 3% by dry weight of soil. In addition, each sample was mixed with FGD gypsum (G) in the proportions of 0, 1, 3, and 5% by dry weight soil, respectively (Table 5). Please note that all the results in this study are the average values from the three samples.

## 3. Results and Discussion

### 3.1. Compaction

**Figure 9:**
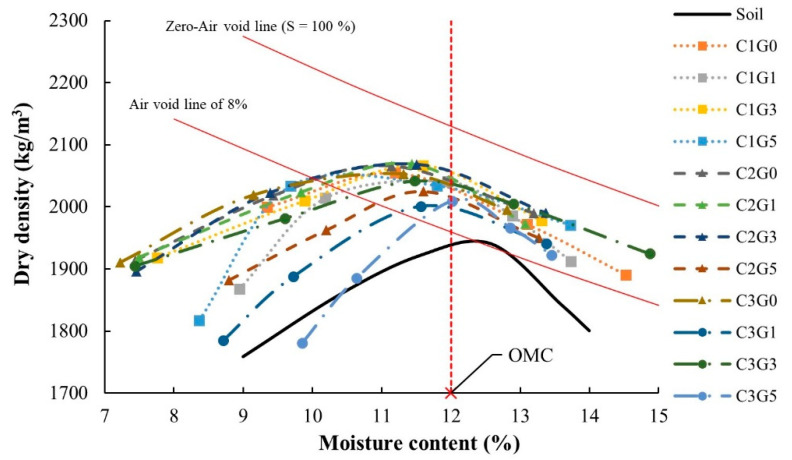
Compaction test results of all samples. **Note**: Soil refers to the substandard lateritic soil used as a benchmark.

The compaction curve referred to the relationship between the dry density, and the moisture contents of a soil sample and is generally used to represent the compaction characteristics of all soil–cement–FGD gypsum mixtures in this study. In Figure 9, the OMCs of all mixes are in between 11.20 and 12.19%, all of which are lower than that of the substandard lateritic soil. Furthermore, as expected, lower OMCs and higher MDDs were observed for all soil–cement–FGD gypsum mixtures compared to the soil. This lower OMC–higher MDD characteristic of the study mixtures was in agreement with previous research [65,66]. The compaction curves of the soil–cement–FGD gypsum mixtures were examined. Significant findings were as follows:

- Adding cement and FGD gypsum to the substandard lateritic soil altered its compaction characteristics as indicated by a higher OMC and a lower OMC (Figure 9). The cause of the lower OMC–higher MDD characteristic was that some of the water in the mixture was consumed by the hydration reaction between cement, FGD gypsum, and water, which yielded solid cementitious products, the generation of which results in more solids in the mixture system, and these cementitious products occupied available void spaces in the mixture system. Consequently, a less porous matrix of the soil–cement–FGD gypsum mixture was obtained, leading to fewer void spaces for water to occupy and lower OMC characteristics of the mixture. Furthermore, those solid cementitious products have a higher specific gravity which led to a much larger overall specific gravity of the mixture compared to the substandard lateritic soil. Therefore, the soil–cement–FGD gypsum mixtures provided a higher MDD than that of the reference soil.
- The soil–cement–FGD gypsum mixtures which were compacted at the larger OMC conditions (the wet-side compaction) showed much higher dry densities compared to those of the substandard lateritic soil. At the MDD, the air void is equal to 8% (see the air void line of 8% in Figure 9) for the reference soil, but all study mixtures demonstrated noticeably fewer air voids. Apart from more solid fractions caused by the new cementitious products in the mixture system, the leftover cement, and FGD gypsum after the hydration reaction assisted in filling available air voids in the mixture system, which led to higher dry densities in the wet-side compaction. This would indicate the addition of a cement–FGD gypsum mixture to the soil provided the lower moisture sensitivity characteristics of a geomaterial.
- A very narrow range (11.20–12.19%) of OMC values was derived for the soil–cement–FGD gypsum mixtures, as seen in a series of compaction curves in Figure 9. This almost identical OMC value caused the presence of Ca^2+^ in the soil–cement–FGD gypsum matrix, which resulted in a reduction of repulsive forces between clay particles due to Ca^2+^ absorption on the surfaces of clay particles in the system. Therefore, the formation of a new edge-to-face arrangement of platy-shaped particles of clay existed [67]. For this unique clay particle arrangement, the unchangeable liquid limit phenomenon of the soil–cement–FGD gypsum mixtures would be expected. This phenomenon can govern a relatively constant OMC value of the mix, based on the findings of [68,69]. They reported that for the clayey soil, its liquid limit was a governing factor in controlling its OMC value.

As a result, an OMC of 12% was used to prepare test samples for all tests in this study.

### 3.2. Strength Development

**Figure 10:**
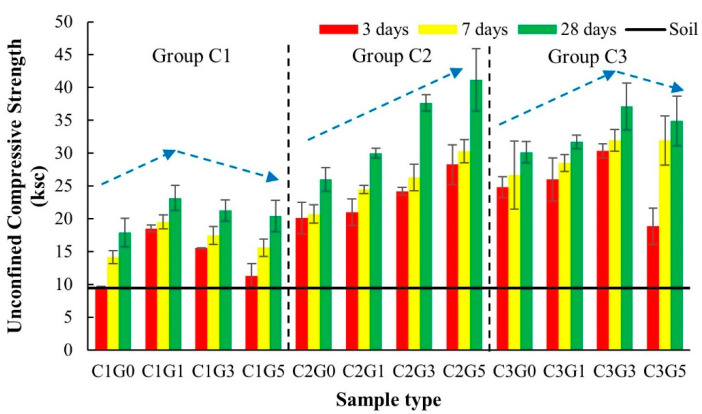
Unconfined compressive strength of the soil–cement–FGD gypsum mixtures.

Test results of the mixes through UCS values are summarized in Table 6 and illustrated in Figure 10. The values of CBR and the CBR index of the target mixes are also presented in Figure 10. Important findings in the strength development and the moisture sensitivity of the mixes are described as follows.

#### 3.2.1. Strength Development Concerning Cement Content, FGD Gypsum Content, and Curing Period

The effects of cement on the strength development of the mixture were considered by three contents of 1, 2, and 3% by dry weight of soil, namely Groups C1, C2, and C3, respectively, as shown in Figure 10. Based on the UCS test results in Figure 10, the higher cement contents yielded higher UCS levels for all mixes. Additional cement added to the mixes increased the UCS value. A series of UCS test results revealed that Group C3 had a higher UCS level than Groups C2 and C1, and Group C2 had a higher UCS level than Group C1, respectively. The strength development of the cement-stabilized soil results from the main solid cementitious compounds of C–S–H was generated from the hydration reaction between cement and water. Regarding the strength development with respect to the UCS of the soil–cement material by varying cement contents, the different zone concept was proposed following previous research work [70,71,72,73]. The soil–cement strength development can be divided into three zones; (1) soil–cement interaction zone (cement content of approximately 0–20%), (2) transitional zone (cement content of roughly 20–30%), and (3) cement–soil interaction zone (cement content of more than 30%). For the cement contents of 1–3% used in this study, the mixtures could be categorized in the soil–cement interaction zone. Within this zone, for a clay cluster in soil, cement had a role in welding water-filled spaces in a clay matrix with inherent cementitious characteristics. As a consequence, the soil–cement interaction zone was established. Also within this zone, interactions among water, clay particles, and cement particles, which have a particulate manner in nature, took place by hydration reactions and the physicochemical actions from cement with water and clay with water, respectively. Therefore, the continuity of a hardened cement paste in which soil particles were embedded existed, as a result of which a higher cement content caused more hardening continuity among soil clusters in the soil–cement matrix because the increase of cement led to higher UCS characteristics.

FGD gypsum content did affect strength development (Figure 10). Adding more FGD gypsum (1–5% of dry soil by weight) generated two patterns in the strength development trends. A rise in UCS values peaked at a certain cement content and then declined with additional FGD gypsum content. Such trends of strength development were observed for Groups C1 and C3. For Group C2, a gradual rise in UCS values over a more FGD gypsum rate was recorded. However, based on the results shown in Figure 10, adding FGD gypsum into a soil–cement mixture resulted in better gains in compressive strength compared to the traditional soil–cement material.

For the curing time, the more extended the period, the higher the recorded UCS values for all mixtures in this study. The strength development through UCS values against curing time indicated and confirmed the on-going chemical reactions for all mixtures within the curing times of 3, 7, and 28 days in this study (Figure 10).

#### 3.2.2. Strength Increase Rate (SIR)

**Figure 11:**
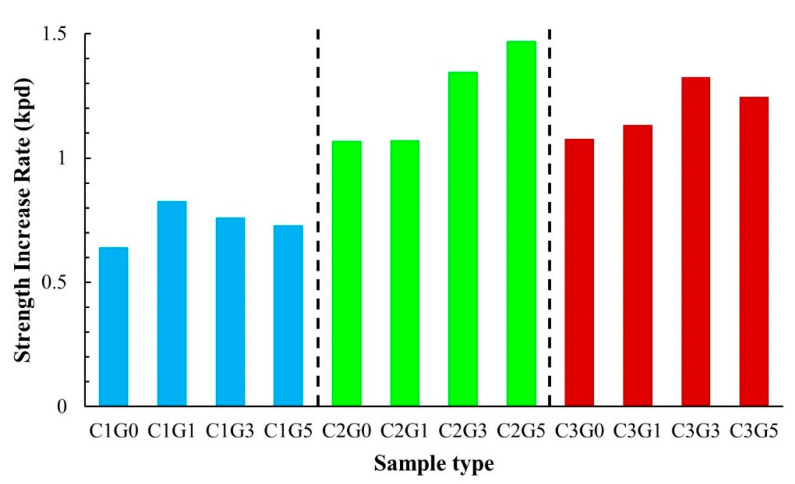
Strength increase rate (SIR) of all mixtures in this study.

C2G5 provides the highest strength increase rate (SIR) of all the samples (Figure 11). Overall, the SIR range was 0.6–1.4 ksc per day. Group C2 demonstrated a higher SIR than Groups C1 and C3, respectively. In addition, when the baseline mixtures of C1G0, C2G0, and C3G0 were considered, adding FGD gypsum to the soil–cement–FGD gypsum mixes tended to show higher SIRs than those of the baseline materials.

#### 3.2.3. Strength Development with Respect To the Water to Cement and FGD Gypsum (w/(c + g)) Ratio

**Figure 12:**
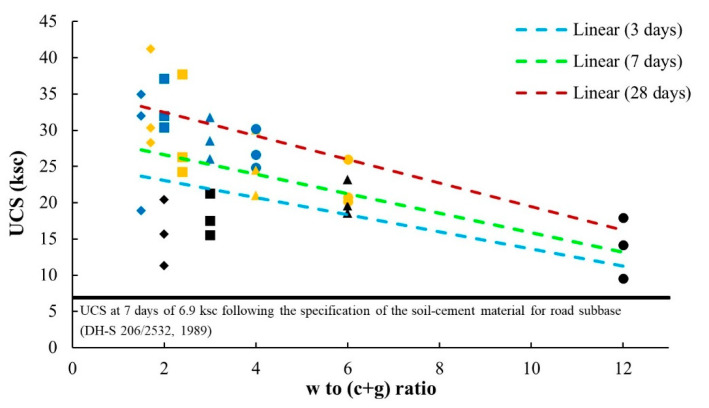
Unconfined compressive strength vs w/(c + g) ratio. **Note**: Line color: light blue, green, and dark red represent 3, 7, and 28 days, respectively. The color of shapes: dark, orange, and blue represents C1, C2, and C3, respectively. Shape: circle, triangle, square, and diamond represent G0, G1, G3, and G5, respectively.

The w/c ratio is a crucial factor in concrete technology since the strength of concrete crucially depends upon such the w/c ratio [74]. Therefore, a newly defined parameter of the water to cement and FGD Gypsum (w/(c + g)) ratio was established in this study. This w/(c + g) ratio was used to capture the strength characteristics of the soil–cement–FGD gypsum mixes relative to the influential parameters of water content, cement content, and FGD-Gypsum content. The trend of the w/c ratio and the compressive strength of concrete were inversely related and the reduction of the w/(c + g) ratio tended to increase the strength of the soil–cement–FGD gypsum mixtures (Figure 12). Furthermore, in Figure 12, UCS values of all mixes in the study were well above 6.9 ksc, the UCS values at seven days, which is based on the specification of the UCS value of the soil–cement material for the road subbase [75].

### 3.3. Moisture Sensitivity

**Figure 13:**
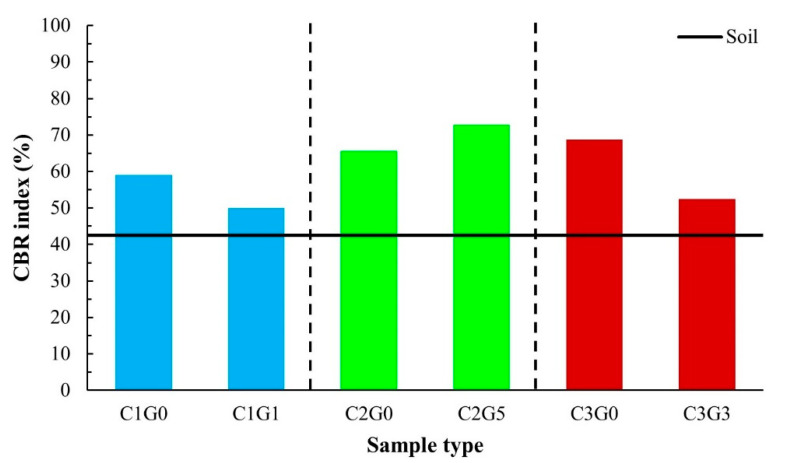
CBR indexes of the target mix in this study.

In this study, the moisture sensitivity of the target mixes was evaluated through the CBR index following Equation (1). The CBR index is not a general parameter indicating the moisture sensitivity characteristics of road base materials; it described the difference in CBR values as a material bearing resistance between relatively dry (unsoaked) and wet (soaked) conditions. C1G1 and C3G3 had the highest USC values in Group C1 and Group C3, respectively, and demonstrated lesser CBR index values than those of C1G0 and C3G0. These lower CBR index values would indicate higher moisture sensitivity of the mixture with the inclusion of FGD gypsum (Figure 13). However, for Group C2, C2G5 had the highest UCS, and SIR also provided a higher CRB index than that of C2G0 and all target mixes. Based on the results shown in Figure 13, that would imply that relatively high UCS values (e.g., C1G1 and C3G3) of the soil–cement–FGD gypsum mixes cannot guarantee a sound moisture sensitivity. Only the addition of a proper amount of FGD gypsum could assist the moisture resistance of the mixture, and C2G5 is the case in this study.

## 4. Roles of FGD-Gypsum Added in the Soil–cement–FGD Gypsum Mixture through SEM Analyses

UCS results illustrated the strength development of the soil–cement–FGD gypsum mixes corresponding to cement content, FGD gypsum content, and curing time (Figure 10). This strength development indicated the effect of the hardening generated from the chemical reactions of cement, FGD gypsum, and water. As was recognized, the well-known theory of conventional cement hydration exists. This theory described the mechanism of how Portland cement compounds of alite or tricalcium silicate (C_3_S), Belite (C_2_S), Aluminate (C_3_A), and Ferrite (C_4_AF) react with water in association with gypsum (CaSO_4_·2H_2_O) to generate the cementitious compounds like C–S–H, calcium hydroxide (CH), ettringite (AFt), and monosulfate (AFm). Gypsum is generally added during cement production as a retarding agent to delay the setting time of the cement paste for workability purposes. In this section, the roles of FGD-gypsum added in the soil–cement–FGD gypsum matrix were explained through the results of SEM analyses.

Starting from the role of the gypsum in cement for the hydration of cement, with the presence of such gypsum, ettringite is formed by the reaction of C_3_A (the majority compound in Portland cement), gypsum, and water, as shown in Equation (2) [76,77]. The ettringite, which has a needle-shaped crystal morphology, could occupy available pore spaces together with C–S–H in a cement-based material, which would lead to the strength gain characteristics of such a material. The ettringite can also further react with the rest of the C_3_A in a paste-formed matrix and then, a more stable stage of monosulfate (3CaO·Al_2_O_3_·CaSO_4_·12H_2_O), a flower-shaped crystal, was formed following Equation (3) [76,77].

3CaO·Al_2_O_3_ + 3(CaSO_4_·2H_2_O) + 26H_2_O → 3CaO·Al_2_O_3_·3CaSO_4_·32H_2_O

(Tricalcium aluminate) + (Gypsum) + (Water) → (Ettringite)(2)

3CaO·Al_2_O_3_ + 3CaO·Al_2_O_3_·3CaSO_4_·32H_2_O + 4H_2_O → 3(3CaO·Al_2_O_3_·CaSO_4_·12H_2_O)

(Tricalcium aluminate) + (Ettringite) + (Water) → (Monosulfate)(3)

At this stage, the chemical products of ettringite and monosulfate were obtained by adding gypsum to the cement and mixing it with water. Furthermore, ettringite generally demonstrated better strength enhancement in a cement-based material––in this case, the soil–cement–FGD gypsum mixtures––than in monosulfate [76,78]. Both ettringite and monosulfate are compounds of C_3_A, CaSO_4_, and water with formation dependent upon the ratios of available alumina to sulfate (Al_2_O_3_/SO_4_, the A/S ratio) in the cement-based material. Ettringite is formed following the condition that a sufficient amount of gypsum exists in the hydration reaction system (high sulfate with the low A/S ratio). Moreover, ettringite can further react with gypsum to convert to monosulfate, a more stable state form than ettringite itself, based on the condition of low sulfate with a high A/S ratio [76,79]. Based on the hydration reaction process, with a continuous increase manner of alumina (Al_2_O_3_), ettringite tends to decrease with a corresponding increase in monosulfate by the time corresponding to a fixed amount of gypsum (a higher A/S ratio condition). To maintain enough ettringite in the hardening process to enhance the strength gain of the soil–cement–FGD gypsum mixtures, a relatively low A/S condition is required. By doing this, more gypsum (a higher amount of sulfate) must be added. In this study, more FGD gypsum was added to the soil–cement–FGD gypsum system. With a sufficient amount of water in the system, the added FGD gypsum provided extra sulfate ions to keep the condition of a low A/S ratio, suitable for ettringite formation. Based on the low A/S ratio environment, the strength gain characteristic of the mixtures was observed. However, adding too much FGD gypsum to the hydration system can cause an adverse effect to the strength gain of the soil–cement–FGD gypsum mixtures. With a limited amount of water in the system to dissolve the sulfate ions from the FGD gypsum, in the case of too much FGD gypsum, gypsum dissolution could take place. This dissolution situation may generate a supersaturated gypsum crystallization in the soil–cement–FGD gypsum mixtures. The FGD gypsum structure itself revealed a weaker structure than that of monosulfate and ettringite. Therefore, the strength-reduction mechanism of the soil–cement–FGD gypsum mixtures can be lost in case of too much gypsum added into the mixture system.

In this study, samples having maximum UCS values of each group (C1G1, C2G5, and C3G3) were examined using SEM.

**Figure 14:**
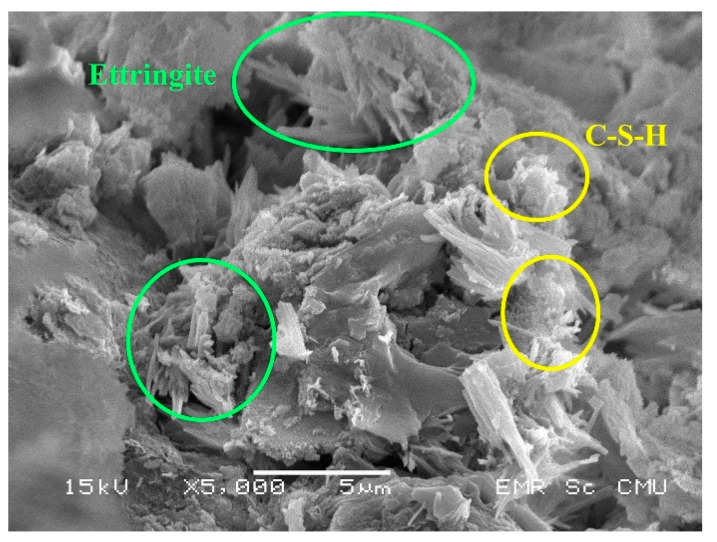
The SEM image of C1G1.

The ettringite and C–S–H gels were observed to confirm the strength gain manner of such a mixture as mentioned above (Figure 14). The further reaction products of monosulfate did not appear with a relatively low gypsum content at this mix of C1G1. However, the strength reduction trend of Group C1 illustrated a range of the FGD gypsum content beyond that of C1G1 (Figure 10). This reduction trend probably resulted from the adverse effect of the excess FGD gypsum, as explained.

**Figure 15:**
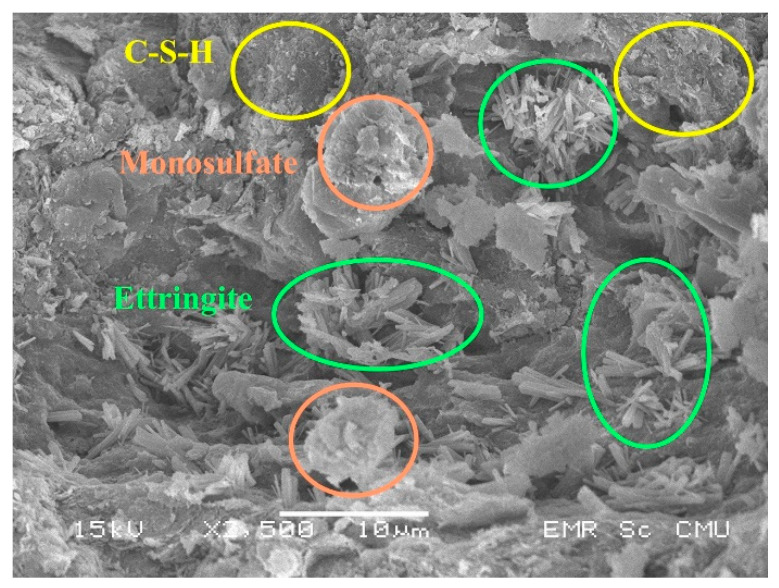
The SEM image of C2G5.

According to Group C2 in Figure 10, a gradual increase in strength exists with no maximum UCS value within the entire range of the mixtures of Group C2. C2G5 demonstrated the highest UCS value in Group C2 and all mixtures in this study. C–S–H, ettringite, and monosulfate are observed in C2G5 to explain its relatively high UCS value (Figure 15). Furthermore, excessive gypsum in the form of supersaturated gypsum, a weaker structure, was not seen. A proper combination among the cement content of 2%, the FGD gypsum contents of 2, 3, and 5%, and active water would play a role in generating C–S–H, ettringite, and monosulfate which are compounds of sound compounds to strengthen the compressive strength of a material. Furthermore, the presence of monosulfate would indicate the interchangeable stage from monosulfate to ettringite under the excessive gypsum environment.

**Figure 16:**
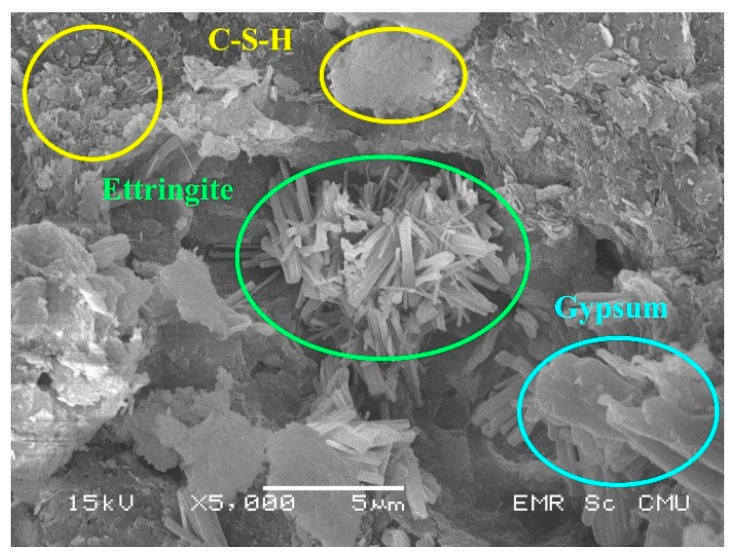
The SEM image of C3G3.

For Group C3, the strength development trend revealed a peak of the UCS value (Figure 10), which was the same as that of Group C1. C3G3 provided the highest UCS value in this group; the strength reduction occurred at the 5% FGD content of C3G5. Apart from C–S–H and ettringite with no monosulfate, unreacted FGD gypsum was not observed in C3G3 (Figure 16). More unreacted FGD gypsum could cause the strength reduction of the mixtures having the FGD gypsum content greater than that of C3G3. In Group C3, with the highest cement content of 3%, an unbalanced amount of active water in the chemical reactions occurred. The water in the mixtures played multiple roles in lubricating soil particles, getting closer, and sharing for the cement hydration during mixing. In the case of C3G5 (the strength reduction), with excessive FGD gypsum, a limited amount of active water to dissolve more sulfate ions existed for some reason. For example, more active water was required to react with more of the 3% cement content, leading, in this case, to less active water in the cement hydration to counteract more FGD gypsum. Active water content for the hydration of the cement would be another influential factor to control the strength gain of the mixture if excessive FGD gypsum were added.

## 5. Application of the Cement–FGD Gypsum Soil Stabilization

Based on the results of this study, FGD gypsum could enhance the use of cement in soil stabilization to obtain a stronger stabilized material. For a target compressive strength of the soil–cement material, adding FGD gypsum into a soil–cement mixture could reduce the cement content of a mix while maintaining such a given compressive strength value.

Based on the w/(c + g) ratio established in this study and the test results of this study, its relationship with UCS values can be expressed through Equation (4).

USC = k_1_ [w/(c + g)] + k_2_ (4)

**Figure 17:**
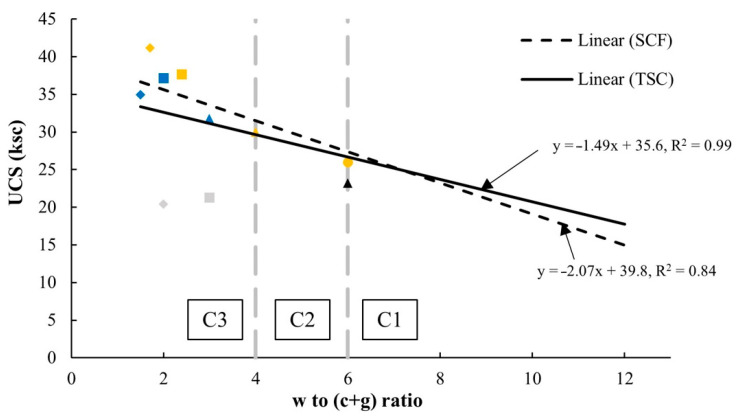
UCS–w/(c + g) relationships. **Note**: Color of shape: dark, orange, and blue represents C1, C2, and C3, respectively. Special color of shape: gray means the value is not used to create the USC–w/(c + g) relationships. Shape: circle, triangle, square, and diamond represent G0, G1, G3, and G5, respectively.

The relationships between the UCS values of the soil–cement–FGD gypsum mixes in this study and the w/(c + g) ratios in Figure 17 are based on the constant w parameter of 12% (the optimum moisture content) and the target cement contents of 1% (C1), 2% (C2), and 3% (C3) as divided zones. For most applications, when a target UCS value was set, according to the linear relationships in Figure 17, a corresponding w/(c + g) to a given UCS value could be found, and then with w = 12% and the correct amount of cement, an optimum amount of FGD gypsum could be calculated for use in a mix. Table 7 illustrates the examples of different proportions of cement and FGD gypsum calculated following given UCS values. This table demonstrates that in the comparison between the traditional soil–cement (TSC) materials (the cement contents of 1, 2, and 3% used in this study) and the soil–cement–FGD gypsum (SCF) materials, adding FGD gypsum reduced the use of cement in a mix to achieve a given UCS value.

## 6. Conclusions

Based on the preliminary examination of the use of FGD gypsum to enhance the effective implementation of the soil–cement mixture in improving poor lateritic soil with a relatively high clay content for road construction purposes in this study, the essential findings of this study can be summarized as follows:

The addition of cement and FGD gypsum into substandard lateritic soil altered its compaction characteristics (see Figure 9). For strength development, higher cement content and higher UCS values were found for all mixes. Furthermore, adding more FGD gypsum (1–5% of dry soil by weight) provided two trends in the strength development: (1) a rise in UCS values peaked at a certain cement content and then declined with greater FGD gypsum content and (2) a gradual increase in UCS values over a more extensive range of FGD gypsum content. For curing time, higher UCS values were observed with more extended curing periods. However, the soil–cement–FGD gypsum mixes could not guarantee sound moisture sensitivity. Only the proper amount of added FGD gypsum could assist the moisture resistance of the mixture. These test results can be well confirmed through the SEM examinations in this study.

The water to cement and FGD gypsum (w/(c + g)) ratio was newly established in this study. This w/(c + g) ratio captured the strength characteristics of the soil–cement–FGD gypsum mixes in relation to the influential parameters of water content, cement content, and FGD gypsum content (Figure 12). The relation between UCS values and the w/(c + g) ratios of the mixtures in this study showed the same trend of the w/c ratio, and the compressive strength of concrete (e.g., the lower the w/c ratio, the higher the compressive strength): the reduction of the w/(c + g) ratio tended to increase the strength of the soil–cement–FGD gypsum mixtures.

For the exercise of how to assign the right amount of FGD gypsum to the soil–cement mixture based on the TSC materials (the cement contents of 1, 2, and 3% used in this study) and the SCF materials, we found that FGD gypsum can reduce the use of cement in a mix to achieve a given UCS value.

## Figures and Tables

**Table 1:** General Requirements for standard subbase materials in tropical areas.

Properties	Thailand: Department of Highways	Brazil: Brazilian National Highway Department [7]		Nigeria: Federal Ministry of Works and Housing	Malaysian: Jabatan Kerja Raya Malaysia	Recommended Criteria Material Selection for Lateritic Soil by Charman’s
	[6]	Heavy Traffic	Light Traffic	[8]	[9]	[10]
California Bearing Ratio, (%)	≥25	≥80	≥60	≥20	≥30	≥25
Percent passing, (%)	-	-	-	No. 200 sieve ≤12	No. 4 sieve ≥45	-
Liquid limit, (%)	≤35	<35	<40	≤25	-	-
Plasticity index, (%)	≤11	<10	<12	≤6	≤12	≤25
Los Angeles abrasion loss, (%)	≤60	<65	<65	-	-	-
Swell, (%)	-	<0.2	<0.2	-	-	-

**Table 2:** Chemical composition of the OPC clinker [29,30].

Compound	Formula (Notation)	Wt.(%)
Tricalcium silicate	3CaO·SiO_2_ (C_3_S)	49–55
Dicalcium silicate	2CaO·SiO_2_ (C_2_S)	20–25
Tricalcium aluminate	3CaO·Al_2_O_3_ (C_3_A)	10–12
Tetracalcium aluminoferrite	4CaO·Al_2_O_3_·Fe_2_O_3_ (C_4_AF)	8
Calcium sulfate dihydrate	CaSO_4_·2H_2_O (CSH_2_)	5
Other Sodium oxide Potassium oxide	Na_2_O (N) K_2_O (K)	1–2

**Table 3:** Properties of FGD gypsum and natural gypsum, adapted from [43,45,46,47].

Property	FGD-Gypsum	Natural Gypsum
Mineral composition	Gypsum, Quartz	Gypsum, Quartz, Dolomite
CaSO4·2H_2_O, (%)	84–99.6	87.1
Specific density, (g/cm^3^)	~2.2	~2.3
Main chemical properties, (%) CaO SO_3_	38 54	59 41

**Table 4:** The physical and engineering properties of lateritic soils.

Properties	A	B	C	DOH Standard (Subbase Materials)	Standard Method
Specific gravity, Gs	-	-	2.86	Not specified	ASTM D854
Passing No. 200 (%)	48	52	64	≤40	ASTM D422
Liquid Limit LL. (%)	30	33	41	≤35	ASTMD4318
Plastic Limit PL. (%)	20	20	19	Not specified	
Plastic Index PI. (%)	10	13	22	≤11	
Classification (as per USCS)	CL	CL	CL	Not specified	ASTM D2487

**Table 5:** Testing scheme.

Purpose	Tests	Moisture	Cement Contents	FGD-Gypsum Contents (% by Dry Weight Soil)	Curing Days (Days)
			(% by Dry Weight Soil)		
Compaction characteristics	Modified Proctor	Various moisture contents to find OMC	0, 1, 2, and 3	0, 1, 3, and 5	-
Strength development	Unconfined compressive strength	OMC	0, 1, 2, and 3	0, 1, 3, and 5	3, 7, and 28
Moisture sensitivity (CBR index)	California bearing ratio		The highest UCS specimens of each cement group at 28 days		28
Microstructure of soil–cement–FGD gypsum	Scanning electron microscope				

**Table 6:** Results of unconfined compression strength test and CBR test.

No.	Specimen Symbol	Cement (% of the Dry Weight of Soil)	FGD Gypsum (% of the Dry Weight of Soil)	Test Result						
				UCS (ksc.)				CBR (%)		CBR Index (%)
				0 Day	3 Days	7 Days	28 Days	Unsoaked	Soaked	
1	Soil	-	-	9.43	-			40	17	42.5
2	C1G0	1	0	-	9.56	14.18	17.94	44	26	59.1
3	C1G1		1		18.54	19.54	23.16	50	25	50.0
4	C1G3		3		15.54	17.46	21.27	-	-	-
5	C1G5		5		11.29	15.63	20.42	-	-	-
6	C2G0	2	0		20.16	20.74	29.92	67	44	65.7
7	C2G1		1		21.01	24.47	30.01	-	-	-
8	C2G3		3		24.21	28.30	37.67	-	-	-
9	C2G5		5		28.26	28.54	41.17	107	78	72.9
10	C3G0	3	0		24.82	26.65	30.17	80	55	68.7
11	C3G1		1		26.04	28.52	31.73	-	-	-
12	C3G3		3		30.36	31.97	37.12	78	41	52.4
13	C3G5		5		18.88	31.95	34.92	-	-	-

**Table 7:** The different proportions of the traditional soil–cement (TSC) materials and the soil–cement–FGD gypsum (SCF) materials corresponding to given USC values.

Process	Target USC	w/c	w/(c + g)	OMC	Cement Content	FGD Gypsum Content
	(ksc)			(% by dry weight soil)		
TSC	20 *	10.5	-	12	1.14	-
SCF		-	9.6		1	0.25
TSC	30 *	3.8	-		3.16	-
SCF		-	4.7		2	0.55
TSC	32.6	2 *	-		6	-
SCF	35.7	-	2 *		3	3

**Note**: the star symbol (*) is used to define (1) target USC or (2) target w/c and w/(c + g) ratio.

## Data Availability

All data and information that support the outputs of this study are available from the corresponding author upon reasonable request.
